# Improving Duty Clinician Decision Making for Medication Side Effects in Patients With Multimorbidity and Polypharmacy: A Quality Improvement Project

**DOI:** 10.1192/bjo.2025.10358

**Published:** 2025-06-20

**Authors:** Tejbir Singh Deol, Wyeshnavi Chitrangan, Rohit Nagaraj, Madhusudan Dalvi, Lucia Laskowski

**Affiliations:** 1 King’s College London, London, United Kingdom; 2 Kent and Medway NHS and Social Care Partnership Trust, Kent, United Kingdom

## Abstract

**Aims:** A large proportion of patients referred to the Shepway Old Age Psychiatry service are multimorbid (3+ health conditions) and have polypharmacy (5+ medications), which can cause a wide range of medication side effects. These side effects, ranging from mild to severe, can compromise patient safety and often result in unnecessary re-entry into the service. This Quality Improvement Project (QIP) aims to improve the management of these patients by implementing a structured triage poster to assist on-call clinicians in deciding whether a patient needs to be readmitted to the old age psychiatric clinic or be referred elsewhere (A&E or GP). The aim of this QIP is to reduce unnecessary referrals, ensure timely intervention for high-risk cases, and optimise appointment allocation within the old age psychiatry service to optimise efficiency in the clinic.

**Methods:** A triage poster was designed and introduced at the old age psychiatry community unit, as a Plan-Do-Study-Act cycle, to standardise management of medication side effects in patients with multimorbidity and polypharmacy. Data on the number of patients readmitted to the service was collected over three weeks prior and three weeks after the implementation of the triage poster. The effectiveness of the poster was assessed by comparing the number of re-admissions and referrals pre- and post-implementation. The mean readmission rates pre- and post-intervention were compared and statistically analysed using a two-sample t-test to assess the impact of the intervention.

**Results:** The mean number of weekly readmissions pre-intervention was 2.33 (SD=1.53). The mean number of weekly readmissions post-intervention was 5.00 (SD=0.00). A two-sample t-test was conducted to compare the means, which showed a statistically significant increase in re-admissions post-intervention (t(4)=−2.92, p=0.043). This demonstrated that the triage poster did not reduce re-admissions and may have caused the opposite intended effect.

**Conclusion:** The implementation of the triage poster was associated with a statistically significant increase in re-admissions to the old age psychiatric community unit. External factors including seasonal patient changes, variations in referral practices, or limited staff training regarding the triage poster may have acted as confounding variables. The short data collection period (three weeks pre- and post-intervention) may not account for realistic variability, which potentially contributed to the observed increase in re-admissions. Further understanding the impact of confounding factors is needed to improve the intervention’s ability to satisfy the QIP’s aim, which is to reduce patient re-admissions related to polypharmacy and multimorbidity.

